# The ferroptosis-mediated domino effect: metabolic crosstalk from intervertebral disc degeneration to spinal deformity and cord injury: a mini review

**DOI:** 10.3389/fnins.2026.1855414

**Published:** 2026-06-19

**Authors:** ZhiYu Zhang, YiBo Dong, Yang Yu, XiaoHong Fan

**Affiliations:** 1 Hospital of Chengdu University of Traditional Chinese Medicine, Chengdu, Sichuan, China; 2 Chongqing Orthopedic Hospital of Traditional Chinese Medicine, Chongqing, China

**Keywords:** blood-spinal cord barrier, ferroptosis, intervertebral disc degeneration, mechanobiology, spinal cord injury, targeted delivery systems

## Abstract

Spinal degeneration, spinal deformity, and spinal cord injury (SCI) are classically managed as discrete biomechanical or neurological entities. However, emerging evidence reveals them as an interconnected pathological continuum. This mini-review introduces the “ferroptosis-mediated domino effect” as the core metabolic driver linking these conditions. The cascade initiates within the avascular intervertebral disc, where aberrant mechanotransduction (e.g., via Piezo1) provokes severe oxidative stress and subsequent ferroptosis, leading to extracellular matrix degradation and structural collapse. The ensuing spinal deformity chronically compresses the spinal microvasculature, disrupting the blood-spinal cord barrier (BSCB) and facilitating localized iron deposition. This chronic ischemic insult generates a metabolically “primed” spinal cord characterized by extreme vulnerability. Upon secondary acute trauma, the sudden influx of heme and labile iron ignites an uncontrollable “ferroptotic storm,” synergizing with neuroinflammation to drive irreversible neural loss. Finally, we evaluate cutting-edge translational interventions—including reactive oxygen species (ROS)-responsive nanoparticles and nanozyme-loaded hydrogels—that offer spatiotemporal precision to halt this pathological crosstalk. By dismantling disciplinary silos, this framework advocates for next-generation, dual-action therapeutic strategies that simultaneously restore biomechanical stability and mitigate metabolic collapse.

## 1 Introduction

Spinal degeneration, spinal deformity, and spinal cord injury (SCI) have conventionally been investigated as isolated clinical entities, largely compartmentalized into distinct biomechanical or neurological disciplines. However, emerging clinical and molecular evidence dictates a paradigm shift: these conditions represent an interconnected pathological continuum (Kumar et al., 2025). The initial biomechanical failure, rather than exerting a mere additive mechanical effect, instigates a profound and devastating cascade of metabolic dysregulation. We propose that the transition from chronic structural deterioration to catastrophic acute neural compromise is fundamentally driven by a shared, progressive metabolic breakdown, creating a lethal cycle of tissue destruction (Gu et al., 2025).

At the epicenter of this pathological cascade lies intervertebral disc degeneration (IVDD). Subjected to extreme hypoxic conditions and aberrant mechanical loading, the avascular intervertebral disc serves as the primary initiation site (Feng et al., 2025). Aberrant mechanical stress vigorously activates mechanosensitive ion channels, most notably Piezo1, which acts as a pivotal mechanotransducer. Piezo1 converts pathological physical forces into destructive biochemical signals, significantly accelerating extracellular matrix (ECM) degradation and cellular senescence. Concurrently, this hostile microenvironment triggers a massive accumulation of reactive oxygen species (ROS) and severe lipid peroxidation, which inexorably drives nucleus pulposus (NP) and annulus fibrosus (AF) cells toward ferroptosis—a distinct, iron-dependent form of programmed cell death. The mechanobiological failure and subsequent ferroptotic cell death synergistically dismantle the disc’s structural integrity (Li et al., 2026).

This structural collapse acts as the critical inflection point, inevitably altering local spinal alignment and generating compensatory deformities. Such biomechanical deformities chronically compress the surrounding spinal microvasculature, inducing a state of sustained localized ischemia (Zeng and Zeng, 2026). Consequently, this chronic hypoxic–ischemic insult progressively disrupts the blood-spinal cord barrier (BSCB). This disruption allows for the abnormal extravasation of red blood cells and macrophages, creating a highly “primed” neural microenvironment characterized by iron dyshomeostasis, severe oxidative stress, and persistent endoplasmic reticulum (ER) stress within the spinal cord tissue (Zhou et al., 2022). When a secondary acute mechanical trauma is superimposed upon this metabolically exhausted and primed spinal cord, the local defense mechanisms are rapidly overwhelmed. The massive release of labile iron, combined with unchecked lipid peroxidation, ignites a “ferroptotic storm.” This aggressive iron-dependent cell death, intricately coupled with ER stress-mediated apoptosis, severely exacerbates local neural tissue damage. Furthermore, these interconnected programmed cell death pathways synergistically release damage-associated molecular patterns (DAMPs), which polarize local microglia and astrocytes into a hyperactive pro-inflammatory phenotype, ultimately driving irreversible neurological deficits and glial scar formation (Fan et al., 2023).

**Figure 1:**
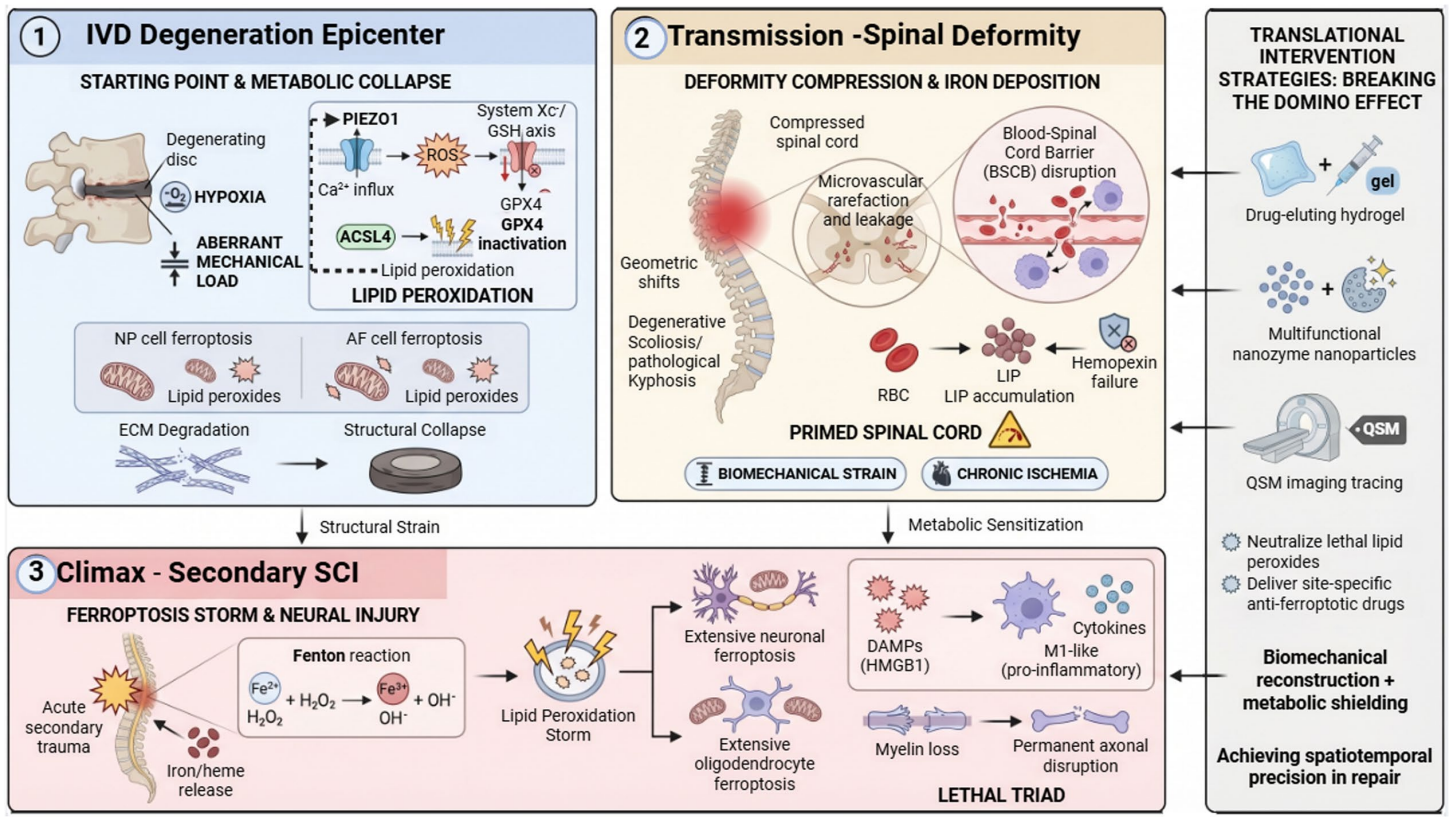
The pathomolecular continuum and translational interventions in the spinal triad. (1) Epicenter—IVD degeneration: Aberrant mechanical loading and hypoxia activate the mechanosensitive ion channel Piezo1, triggering Ca2 + influx and excessive ROS generation. This oxidative stress collapses the System Xc-/GSH antioxidant axis and inactivates GPX4, facilitating ACSL4-driven lipid peroxidation. Crucially, this lipid peroxidation creates a positive feedback loop (indicated by the dashed arrow) that further sensitizes the Piezo1 channel. This vicious cycle ultimately drives nucleus pulposus (NP)/annulus fibrosus (AF) cell ferroptosis, leading to extracellular matrix (ECM) degradation and structural collapse. (2) Transmission and priming stage—Spinal deformity: The resulting spinal deformity (e.g., degenerative scoliosis or kyphosis) imposes chronic mechanical tethering and microvascular compression on the spinal cord. This leads to blood-spinal cord barrier (BSCB) disruption and the extravasation of iron-rich blood components, creating a labile iron pool (LIP). As visually highlighted by the localized red glow, this chronic ischemic and inflammatory insult generates a highly vulnerable “primed” spinal cord with depleted antioxidant reserves. > (3) Climax—Secondary SCI: Upon acute secondary trauma, a massive release of iron/heme ignites a ferroptotic storm via the Fenton reaction. This catastrophic lipid peroxidation causes extensive neuronal and oligodendrocyte death, accompanied by the release of DAMPs (e.g., HMGB1), which polarize local microglia/macrophages into a pro-inflammatory M1-like phenotype, resulting in irreversible myelin loss and axonal disruption. Created with BioRender.com.

Therefore, this review proposes a novel, integrative conceptual framework: the “ferroptosis-mediated domino effect.” By dismantling traditional disciplinary silos, we aim to systematically elucidate the molecular and biomechanical crosstalk linking IVDD-induced structural failure to SCI-associated neural death. Finally, we comprehensively evaluate targeted translational interventions—ranging from Piezo1 modulators to advanced ferroptosis inhibitors and regenerative tissue engineering—that hold immense therapeutic potential to simultaneously restore spinal structural stability and mitigate catastrophic neuroinflammation (Figure 1).

## 2 The epicenter: mechanotransduction and ferroptosis in intervertebral disc degeneration

The intervertebral disc (IVD) resides in a uniquely hostile, avascular, and hypoxic niche, subjected to unrelenting and complex mechanical loading. While physiological loading is indispensable for maintaining extracellular matrix (ECM) homeostasis, pathological mechanical strain precipitates a profound metabolic crisis within NP cells. Emerging evidence identifies the mechanosensitive ion channel Piezo1 as the primary molecular transducer at this mechanical-biochemical interface (Li et al., 2026). Upon activation by aberrant high-magnitude compression or shear stress, Piezo1 undergoes a conformational shift, facilitating a massive influx of extracellular calcium (Ca^2+^). This cationic surge initiates a deleterious signaling cascade that effectively converts physical forces into intracellular destructive events, including mitochondrial dysfunction and the activation of the NLRP3 inflammasome (Li et al., 2026; Chen S. et al., 2026).

Crucially, this Piezo1-mediated calcium overload instigates a lethal accumulation of reactive oxygen species (ROS), pushing the NP cells toward a state of severe oxidative exhaustion (Chen et al., 2025). In this “primed” oxidative environment, the cellular antioxidant defense system—specifically the System Xc−/Glutathione (GSH) axis—is systematically dismantled. The resultant depletion of GSH directly inactivates Glutathione Peroxidase 4 (GPX4), the master rheostat of ferroptotic cell death (Nan et al., 2026). Unlike apoptosis, which follows a controlled proteolytic program, this mechanical-induced ferroptosis is characterized by unchecked iron-dependent lipid peroxidation.

The interplay between Piezo1 activation and ferroptosis creates a “feed-forward” loop: pathological stress-induced ROS leads to lipid peroxyl radical formation on the cell membrane, which further alters membrane fluidity and potentially sensitizes Piezo1 channels to even lower mechanical thresholds (Chen et al., 2023; Zhu et al., 2021). This metabolic-biomechanical crosstalk results in a catastrophic loss of functional NP cells and an accelerated degradation of the proteoglycan-rich matrix. Recent longitudinal analyses further validate that sustained mechanical stress directly upregulates ferroptosis-related gene signatures in disc tissues well before gross structural failure occurs (Wang et al., 2026). Thus, the Piezo1-ferroptosis axis represents the “first domino” in the spinal triad, translating initial structural strain into a self-amplifying cycle of tissue failure.

## 3 Transmission: structural collapse, microvascular compression, and the “primed” spinal cord

The transition from localized intervertebral disc failure to systemic spinal dysfunction occurs through a distinct, two-phase mechanometabolic chain.

Phase 1: Biomechanical Collapse and Spinal Deformity. Initially, the catastrophic loss of structural integrity in the degenerated intervertebral disc dictates a critical shift in spinal alignment (Li et al., 2023; Kim et al., 2023). Advanced IVDD acts as the mechanical tipping point, driving compensatory but pathological geometric shifts such as degenerative scoliosis or kyphosis (Long et al., 2012; Long et al., 2015). This structural failure imposes a sustained, abnormal physical tethering and mechanical compression on the adjacent spinal canal contents (Long et al., 2012).

Phase 2: Microvascular Compression and Metabolic Priming. Following the primary mechanical disruption, the sustained tethering selectively compresses the highly vulnerable spinal microvasculature (Li et al., 2023). This physical constriction precipitates microvascular rarefaction and persistent localized ischemia, effectively translating the mechanical deformity into a severe metabolic crisis (Li et al., 2023; Yao et al., 2022). The chronic hypoxic–ischemic environment progressively dismantles the blood-spinal cord barrier (BSCB), allowing the extravasation of iron-rich blood components (Otasevic et al., 2021; Zhou et al., 2020). This early metabolic priming is strongly supported by emerging clinical quantitative MRI studies. Recent quantitative susceptibility mapping (QSM) analyses of patients with degenerative cervical myelopathy have demonstrated that localized iron deposition and tissue structural damage can be non-invasively tracked (Freund et al., 2024). Crucially, the severity of these advanced QSM metrics shows a strong negative correlation with clinical neurological function, specifically the modified Japanese Orthopaedic Association (mJOA) scores (He et al., 2022). This statistical evidence solidifies the premise that progressive metabolic dyshomeostasis is not merely an incidental histological finding, but a primary driver of functional decline well before an acute secondary trauma occurs.

We define this pathological state as the “Primed Spinal Cord”—a metabolic “pre-crisis” where the neural tissue, though still functionally compensated, resides at the precipice of ferroptotic collapse. It is crucial to distinguish this state from a chronically compressed cord without metabolic sensitization. Purely biomechanical compression may induce transient ischemia and reversible neuropraxia; however, the defining inflection point of a “primed” state is the definitive structural breakdown of the blood-spinal cord barrier (BSCB) (Kim et al., 2023). This disruption facilitates the continuous extravasation of immune cells and iron-rich blood components, triggering widespread microglial activation and establishing a pro-apoptotic molecular signature (Yao et al., 2022). In this sensitized state, the neural tissue is no longer merely compressed; it is metabolically exhausted and possesses a severely diminished capacity to buffer any subsequent oxidative surge or iron overload. This metabolic fragility ensures that when the “domino effect” reaches its climax through a secondary injury, the resulting ferroptotic cascade is both immediate and catastrophic.

## 4 The climax: ferroptotic cascade and neuroinflammatory amplification in secondary SCI

The culmination of the “domino effect” occurs when a secondary acute mechanical trauma is superimposed upon a metabolically sensitized spinal cord. This acute insult precipitates massive intraparenchymal hemorrhage, leading to the catastrophic release of heme and labile iron into the neural environment (Kafura et al., 2025). Unlike the chronic, low-magnitude iron accumulation seen during the “priming” stage, this acute surge overwhelms the sequestering capacity of heme-binding proteins like hemopexin, directly fueling the Fenton reaction and generating an uncontrollable burst of hydroxyl radicals (Kafura et al., 2025; Pelisch et al., 2020).

The resultant oxidative stress triggers a swift and profound suppression of GPX4, the primary guardian against lipid peroxidation. Single-cell RNA sequencing reveals that while certain selenoproteins may exhibit a transient, compensatory rise immediately following injury, they are rapidly superseded by the sustained induction of ferroptosis drivers, most notably acyl-CoA synthetase long-chain family member 4 (ACSL4) (Fan et al., 2025). This enzymatic imbalance leads to the lethal accumulation of lipid peroxyl radicals on neuronal and oligodendrocyte membranes, a process further exacerbated by lysosomal membrane permeabilization and the failure of protective proteins like oxysterol binding protein like 10 (OSBPL10) to maintain membrane integrity (Zhang et al., 2026; Wei et al., 2022; Xue et al., 2026). The shrunken mitochondria with condensed cristae—a hallmark of ferroptosis—signify the irreversible collapse of the neural metabolic framework (Zhang et al., 2026).

Beyond isolated cellular demise, this “ferroptotic storm” acts as a potent orchestrator of neuroinflammation. Ferroptotic neurons and myelin-producing cells release specific DAMPs, such as high-mobility group box 1 (HMGB1), which aggressively polarize local microglia and infiltrating macrophages into a hyperactive pro-inflammatory phenotype (Kafura et al., 2025; Xue et al., 2026; Ryan et al., 2024). This glial activation creates a self-amplifying feedback loop: the secreted pro-inflammatory cytokines further impair the System Xc-/GSH axis, lowering the threshold for ferroptosis in neighboring healthy cells (Fan et al., 2025; Xue et al., 2026; Zhou et al., 2024). Consequently, the initial mechanical injury is translated into a spreading wave of secondary tissue damage, characterized by progressive myelin loss and permanent axonal disruption. This metabolic-inflammatory nexus ultimately solidifies the transition from structural instability to irreversible neurological deficit.

## 5 Precision interventions: disrupting the domino effect via advanced delivery systems

The clinical management of the “spinal triad” necessitates a shift from passive decompression to active, spatiotemporal regulation of the pathological microenvironment. Traditional systemic administration of ferroptosis inhibitors is often thwarted by low bioavailability and the restrictive nature of the blood-spinal cord barrier (BSCB). To dismantle the “domino effect,” emerging translational strategies utilize stimuli-responsive biomaterials to achieve site-specific delivery and multi-modal repair (Zhou et al., 2024; Fan et al., 2022).

Nanotechnology offers a sophisticated platform for targeting the metabolic climax of the triad. Recent breakthroughs include ROS-responsive nanoparticles designed to deliver ferroptosis inhibitor prodrugs (e.g., Ferrostatin-1) specifically to the injured spinal parenchyma (Zhou et al., 2024; Xu et al., 2026). These “smart” nanocarriers exploit the high oxidative stress characteristic of the “ferroptotic storm,” releasing their cargo only upon encountering elevated ROS levels. This approach not only neutralizes lethal lipid peroxides but also enhances the survival and integration of co-delivered mesenchymal stem cells (MSCs), thereby facilitating neural circuit reconstruction (Zhou et al., 2024; She et al., 2025). Furthermore, natural products with potent anti-ferroptotic properties are being encapsulated into targeted nanoplatforms to enhance their therapeutic index and achieve sustained neuroprotection (She et al., 2025; Fan et al., 2025).

Complementing these nano-strategies, multifunctional hydrogels serve as both structural scaffolds and biochemical modulators. For patients with combined spinal deformity and chronic compression, conductive hydrogels can be deployed to bridge the structural gap while mimicking the spinal cord’s endogenous electrical signaling environment (Du et al., 2025). Advanced formulations incorporate “nanozymes,” such as Tellurium-based clusters, which possess multi-enzyme-like activities to persistently scavenge ROS and inhibit the ferroptotic cascade within the spinal microenvironment (Meng et al., 2024; Li and He, 2024). These hydrogels can be tailored with self-healing and injury-responsive properties, allowing for minimally invasive delivery via the epidural or subarachnoid space (Fan et al., 2022; Li and He, 2024). By integrating structural stabilization with the targeted inhibition of ferroptosis, these bioengineered systems provide a comprehensive toolkit to halt the progression from intervertebral disc failure to irreversible neurological decline.

### 5.1 Translational challenges in *in vivo* delivery

Despite the immense preclinical promise of smart biomaterials, several formidable translational hurdles must be addressed before clinical application. First, BSCB Penetration and Targeting Efficiency: Systemic administration of nanocarriers often exhibits poor accumulation in the spinal parenchyma due to the restrictive BSCB. Future designs must incorporate active targeting ligands—such as matrix metalloproteinase (MMP)-responsive peptides that specifically bind to injured microvascular endothelium—to enhance site-specific accumulation across the disrupted barrier (Li et al., 2026). Second, The Delivery Time Dimension: The “ferroptotic storm” is an acute event that peaks within hours to days post-secondary injury. Hydrogel degradation and drug-release kinetics must be exquisitely calibrated to match this narrow therapeutic window, providing an initial burst release to quench the acute lipid peroxidation, followed by sustained release to suppress secondary inflammatory waves. Finally, Long-term Biosafety: The utilization of metal-based multi-functional “nanozymes” raises significant concerns regarding long-term heavy metal toxicity, unpredictable intracellular degradation pathways, and neurological clearance mechanisms (Yang et al., 2025). Comprehensive *in vivo* pharmacokinetic profiling will be imperative for safe human translation.

## 6 Summary and outlook

### 6.1 Schools of thought and current controversies

A major point of contention involves the dual nature of ferroptosis in early spinal pathology. While most evidence labels ferroptosis as a purely detrimental driver of nucleus pulposus (NP) cell loss and secondary SCI, some schools of thought suggest that early-stage ferroptosis might serve as a “protective clearing” mechanism to eliminate terminally stressed cells before they trigger systemic inflammatory leakage (Song et al., 2026; Shi et al., 2026).

Furthermore, characterizing the crosstalk between ferroptosis and other regulated cell death (RCD) pathways presents a significant conceptual conundrum. In the complex microenvironment of SCI, these pathways do not operate in isolation. While apoptosis typically drives delayed, secondary neuronal and oligodendrocyte loss through controlled, caspase-dependent proteolytic cascades, and pyroptosis amplifies acute neural damage via inflammasome-mediated membrane pore formation and robust cytokine release, ferroptosis is uniquely characterized by rapid, iron-dependent lipid peroxidation that fundamentally destroys membrane integrity (Ni et al., 2026). Adding to this complexity is emerging evidence on cuproptosis—a copper-dependent mitochondrial collapse—which suggests that metal-ion dyshomeostasis is not limited to iron alone. The dynamic interplay among iron-driven lipid peroxidation, caspase-mediated apoptosis, inflammatory pyroptosis, and copper-induced proteotoxic stress likely forms a synergistic, highly lethal ‘PANoptosis’ network that ultimately dictates the severity and irreversibility of neural loss (Ni et al., 2026; Liu et al., 2026; Chen W. et al., 2026). Specifically, the molecular crosstalk among these pathways creates a highly integrated cell death network known as PANoptosis (Xu et al., 2026). For instance, the massive accumulation of reactive oxygen species (ROS) and lipid peroxides during early ferroptosis serves as a potent upstream signal that can activate inflammasomes, directly linking oxidative stress to pyroptosis and subsequent neuroinflammation. Concurrently, severe lipid peroxidation permanently damages mitochondrial membranes, promoting the release of cytochrome c and triggering caspase-dependent apoptosis (Qian et al., 2026). Therefore, the progression of secondary SCI is not driven by a singular cell death executioner, but rather by the synergistic activation of these pro-inflammatory programmed cell death pathways within the multi-protein PANoptosome complex (Xu et al., 2026).

Additionally, the primary source of the labile iron pool (LIP) during the “priming” stage remains debated. Is it predominantly derived from occult micro-hemorrhage or from the metabolic dysfunction of resident macrophages and microglia failing to sequester iron via the ferritin-ferritinophagy axis? (Chen W. et al., 2026). Resolving this will be crucial for determining whether vascular stabilization or metabolic reprogramming should be the priority in early intervention.

### 6.2 Current research gaps

Crucially, it must be acknowledged that the “ferroptosis-mediated domino effect” proposed in this review remains a largely theoretical conceptual framework synthesized from disparate clinical and molecular observations. A fundamental limitation in validating this continuum is the stark absence of comprehensive animal models (Zhao et al., 2026; Yu et al., 2025). Currently, the majority of *in vivo* studies rely on isolated paradigms—either acute contusive SCI models or static, chemically-induced IVDD models. These isolated models fundamentally fail to recapitulate the longitudinal, multi-stage pathophysiological transition from initial disc degeneration to progressive spinal deformity, and ultimately to secondary spinal cord sensitization and injury (Mai et al., 2026). To definitively validate this framework, developing composite, bio-fidelic animal models is an urgent prerequisite. We propose a “two-stage mechanical-to-metabolic” in vivo paradigm. Phase 1 (The Priming Stage): A chronic intervertebral disc degeneration and deformity model can be established in rodents using a combined approach of percutaneous needle puncture of the disc and subsequent bipedal standing induction (to simulate axial loading and progressive kyphosis) (Ao et al., 2019). Alternatively, placing a slowly expanding water-absorbing polymer in the epidural space can precisely mimic the progressive mechanical tethering and gradual blood-spinal cord barrier (BSCB) breakdown (Ijima et al., 2017). Phase 2 (The Climax Stage): Once quantitative MRI or behavioral assessments confirm the establishment of a localized “sensitized” microenvironment (iron deposition and sustained local inflammation), a standardized acute contusion injury—or an acute decompression-induced ischemia–reperfusion stress—is superimposed exactly at the pathologically primed segment (Yang et al., 2015). This “two-hit” methodology, combining chronic structural priming with an acute mechanical or vascular climax, is clinically and experimentally highly relevant (Kubota et al., 2026). This sequential in vivo paradigm will finally allow researchers to continuously track the longitudinal spatiotemporal propagation of ferroptotic signals from the collapsed disc to the irreversibly damaged neural tracts (Zhang et al., 2025).

### 6.3 Potential future developments

The future of spinal triad management lies at the intersection of high-resolution molecular mapping and bio-intelligent engineering. First, the application of spatial transcriptomics and metabolomics is imperative to draw the “metabolic atlas” of the spinal microenvironment, allowing us to see exactly how ferroptotic signals propagate across the disc-bone-cord interface (Ni et al., 2026; Liu et al., 2026). Second, the clinical translation of Quantitative Susceptibility Mapping (QSM) holds immense promise. By utilizing QSM to non-invasively track iron deposition in the spinal cord, we can potentially establish a predictive biomarker for neurological decline in patients with chronic deformity (Mai et al., 2026). Finally, we envision the development of “Dual-Action Smart Implants.” These next-generation devices, such as drug-eluting interbody fusion cages or antibody-loaded collagen scaffolds, would provide the necessary biomechanical support to correct deformity while simultaneously releasing site-specific ferroptosis inhibitors or “nanozymes” to quench the metabolic storm (Zhao et al., 2026; Sicard et al., 2024). By integrating structural restoration with metabolic shielding, we can effectively halt the domino effect and usher in a new era of patient-centered spinal care.
